# A Review of Human Immunodeficiency Virus and Hepatitis B Virus Co-Infection in Botswana

**DOI:** 10.3390/v18050523

**Published:** 2026-04-30

**Authors:** Linda Mpofu-Dobo, Kebaneilwe Lebani, Jason T. Blackard, Sikhulile Moyo, Motswedi Anderson, Simani Gaseitsiwe

**Affiliations:** 1 Botswana Harvard Health Partnership, Gaborone Private Bag BO320, Botswana; ldobo@bhp.org.bw (L.M.-D.); smoyo@bhp.org.bw (S.M.); manderson@bhp.org.bw (M.A.); 2 Department of Biological Sciences and Biotechnology, School of Life Sciences, Botswana International University of Science and Technology, Palapye Private Bag 16, Botswana; lebanik@biust.ac.bw; 3 Department of Internal Medicine, University of Cincinnati College of Medicine, Cincinnati, OH 45267, USA; jason.blackard@uc.edu; 4 Department of Virology, Sefako Makgatho Health Sciences University, Pretoria 0204, South Africa; 5 Department of Immunology and Infectious Diseases, Harvard T.H. Chan School of Public Health, Boston, MA 02115, USA; 6 Department of Pathology, Division of Medical Virology, Faculty of Medicine and Health Sciences, Stellenbosch University, Cape Town 7505, South Africa; 7 Africa Health Research Institute, Private Bag X7, Congella, Durban 4013, South Africa; 8 The Francis Crick Institute, London NW1 1AT, UK

**Keywords:** hepatitis B virus (HBV), human immunodeficiency virus (HIV), HIV-HBV co-infection, sub-Saharan Africa

## Abstract

Hepatitis B virus (HBV) remains a leading cause of chronic liver disease worldwide, contributing to cirrhosis and hepatocellular carcinoma. Sub-Saharan Africa accounts for an estimated 68% of incident HBV infections, where co-infection with human immunodeficiency virus (HIV) is common and associated with poorer clinical outcomes. In Botswana, limited HBV screening and the absence of established HBV management guidelines persist despite reported HIV-HBV co-infection rates ranging from 1.1% to 10.6%. This scoping review aimed to summarise existing research on HBV and HIV-HBV co-infection in Botswana and assess clinical and policy implications. Following PRISMA methodology, searches were conducted across PubMed, Google Scholar, Semantic Scholar, and Consensus databases. Thirty eligible peer-reviewed studies were identified and evaluated for prevalence data, virological characteristics, genotypes, mutations, treatment outcomes, vaccination programs, and the availability of guidelines. Findings indicate intermediate-to-high HBV and HIV-HBV disease burden, substantial occult HBV infection, and gaps in diagnostic and preventive practices. The lack of routine screening, deficient infant birth-dose and adult vaccination, and established treatment pathways likely increase the risk of HBV-associated morbidity and mortality. Strengthened public health interventions, including expanded testing, enhanced vaccination coverage, and prevention of mother-to-child transmission strategies, are recommended to improve disease control and clinical outcomes in Botswana.

## 1. Introduction

There are increasing efforts to improve the management of viral hepatitis globally. In this accord, the World Health Organisation (WHO) has set elimination targets and guidelines for hepatitis B and hepatitis C by 2030 [1]. Key to this elimination is data indicating the potential benefits of hepatitis B virus (HBV) prevention, which could avert approximately 25 million new infections and 8 million deaths worldwide [2]. Prevention efforts are focused on improving access to vaccines, increasing diagnosis coverage, as well as providing timely access to existing, effective treatment. These efforts are essential to help prevent devastating liver-associated sequelae such as cirrhosis and hepatocellular carcinoma (HCC) [3,4], which can develop in 3% [5] of those living with HBV infection per year and 10–25% of people living with HBV over a lifetime [6,7]. HBV contributes to 83% of deaths related to viral hepatitis, while the remaining 17% of deaths are attributed to hepatitis C virus (HCV) [4].

In Botswana, as with other countries with a high burden of human immunodeficiency virus (HIV) (20.8% prevalence as of 2021) [8], co-infection with HBV is of particular concern due to shared routes of transmission and the potential effect on clinical outcomes. Thus, the majority of epidemiological and virological research into viral hepatitis in Botswana has been in the context of HIV-HBV co-infection [3,4]. This review will focus on HBV-related research conducted thus far in Botswana and provide insights into future work to address the burden of HBV on the country’s health system. Table 1 presents the key HBV background information necessary for this review.

### 1.1. Global Epidemiology of the Hepatitis B Virus and the Human Immunodeficiency Virus

HBV is known as a silent epidemic due to the large number of people living with HBV who are unaware of their infection status until they develop overt symptoms [24]. The epidemiology of HBV and HIV-HBV has been discussed extensively elsewhere. A summary of the epidemiology of HBV and HIV-HBV co-infection globally, in Africa, and in Botswana is presented in Table 1.

### 1.2. Diagnosis of HBV and HIV

HBV is diagnosed by measuring the presence or absence of HBV antigens and antibodies produced against the virus. Some countries also measure HBV DNA levels in the blood. Testing strategies and algorithms are varied and have been detailed in prior literature. Table 1 contains a brief summary and relevant sources of information.

### 1.3. Vaccines and Treatment of HBV and HIV

The HBV subunit vaccine has been part of routine childhood vaccination schedules in a growing number of countries since the WHO recommendation was established in 1991 [25,26]. Adult vaccination is also encouraged, particularly for those at increased risk of acquiring HBV. Table 1 highlights key information on vaccination, along with relevant sources for deeper insight.

For the treatment of HBV, the WHO recommends nucleoside reverse transcriptase inhibitors (NRTIs), particularly tenofovir (tenofovir disoproxil fumarate [TDF] or tenofovir alafenamide [TAF]) and entecavir (ETV) [22]. Further information on treatment and prophylaxis can be found in Table 1.

### 1.4. Complications of HIV-HBV Co-Infection

The 2024 WHO hepatitis report states that “In the absence of treatment, HIV co-infection profoundly affects many aspects of the natural history of HBV infection, such as more rapid progression to cirrhosis and HCC, higher liver-related mortality, higher rates of chronicity after acute HBV infection, increased rates of reactivation and rates of occult hepatitis B (OBI), and reduced treatment response compared with people without HIV co-infection” [4].

The accelerated progression of liver disease, including cirrhosis, HCC, and end-stage liver failure, as well as increased mortality (liver-related and all-cause) are thought to be caused by HIV’s suppressive effect on the immune system [27]. This poses a challenge, with HIV-HBV co-infection exacerbating the damaging effects of HBV on the liver. Although this is the case, cART treatment for HIV with an agent active against HBV, such as tenofovir, has been found to be beneficial for HIV-HBV co-infection [21]. Co-infected individuals also benefit from liver-related monitoring [21].

The hepatotoxic effects of cART are a consideration prior to initiating treatment in HIV-HBV co-infected individuals. Tenofovir—recommended by WHO and included in HIV cART—does not seem to cause hepatotoxicity in people with healthy livers, although certain classes of antiretroviral (ARV) drugs are associated with hepatotoxicity, as described in an article by Otto et al. [28]. Immune Reconstitution Inflammatory Syndrome (IRIS) is a potential complication after initiating cART in PLWH.

IRIS is characterised by an aberrant hyperinflammatory response and can cause hepatic flares of elevated liver enzymes compounding the hepatic impairment caused by HBV. These IRIS-induced hepatic flares can lead to acute liver failure [29].

In addition to liver-related complications, non-liver-related effects of HIV-HBV co-infection include the financial implications of monitoring both infections. HBV viral load testing is often not performed, despite being important for disease monitoring and the determination of treatment timing. This is primarily due to the huge cost associated with viral load testing. Other tests needed to monitor both infections and treatment also require a large financial commitment. Another non-liver-related effect of HIV-HBV co-infection is the psychosocial effect of living with HIV and HBV. Psychosocial effects mainly revolve around the stigma that exists in relation to both HIV and HBV. Significant stigma exists due to the association of HIV and HBV infection with sexual promiscuity and intravenous drug use; more so if one is living with both infections [30].

Paradoxical to the negative effects of HIV-HBV co-infection on HBV disease progression, HIV infection has thus far not been found to be worsened by concomitant HBV infection [12,27].

## 2. Methods

### 2.1. Review Protocol

A review protocol was not registered for this review article.

### 2.2. Search Strategy

**Figure 1:**
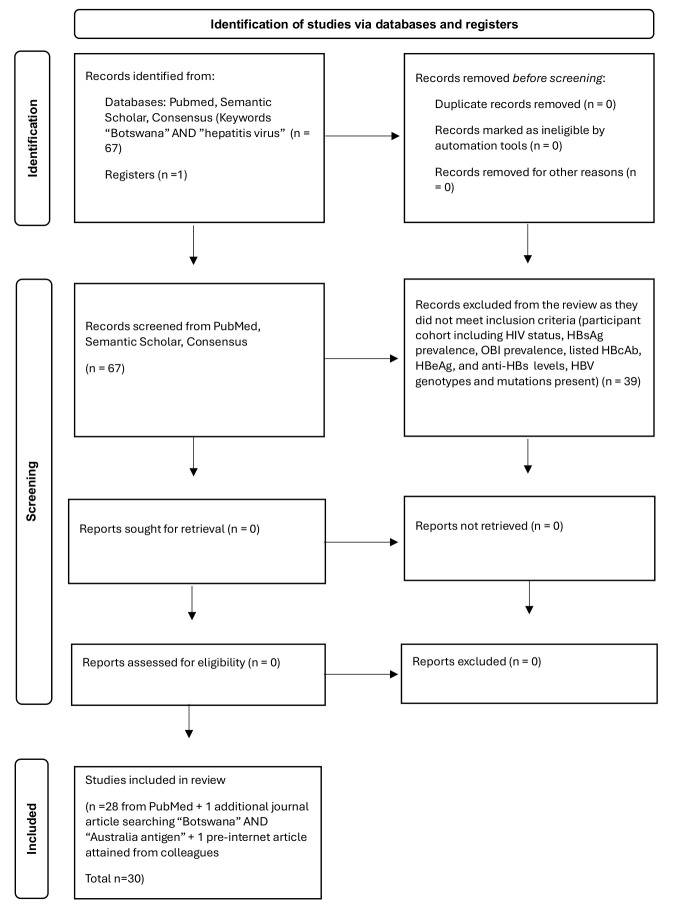
Flowchart depicting process for identifying articles to be included in the review, according to the Preferred Reporting Guidelines for Systematic Reviews and Meta-Analyses (PRISMA) [31].

A search conducted as per the Preferred Reporting Guidelines for Systematic Reviews and Meta-Analyses (PRISMA) [31], with the keywords “Botswana” AND “hepatitis virus” with no limitations on time period, was performed on 2 April 2024 in the National Center for Biotechnology Information (NCBI) PubMed online platform. The same search was conducted via Google Scholar, Semantic Scholar and Consensus online platforms to find additional relevant articles that were not found via PubMed. Additionally, a search was performed using the keywords “Botswana” AND “Australia antigen”, the former name of HBsAg, with a timeline limit of publication before 2006 on the same online platforms listed above. A repeat of the searches on all online platforms listed was performed on 2 October 2025. The process is illustrated in a flowchart in Figure 1.

### 2.3. Study Selection Criteria

Articles found from searches were reviewed and only those that specifically related to hepatitis B in Botswana, written in English, and peer-reviewed were included for this review.

### 2.4. Data Extraction

From the articles selected for inclusion, the following data were extracted where available: risk category, HIV status, HBsAg prevalence, OBI prevalence, anti-HBc, HBeAg, and anti-HBs levels, HBV genotypes, and viral mutations. Pertinent article information was summarised in Microsoft^®^ Excel for Mac version 16.107.1, including the publication year, authors, HBV serological and viral load results, as well as mutations and clinical outcomes.

### 2.5. Process for Designing Recommendations on Improvement of HIV-HBV Management in Botswana

Recommendations for improvement of care for those living with HIV and HBV were designed based on reviewing HBV and HIV-HBV co-infection management guidelines, practices and data, where available, in Botswana. The information gathered was then compared to international guidelines and best practices on HBV and HIV-HBV co-infection management set by the WHO. Differences observed in Botswana that were determined to be causing deficiencies in care of those living with HIV and HBV were utilised as the basis for recommendations on improvement of care.

## 3. Search Results

From the search of PubMed using the search terms “Botswana” AND “hepatitis virus”, 50 articles were initially retrieved on 2 April 2024. The further search on 2 October 2025 retrieved 17 additional articles, bringing the total to 67. The search of the terms “Botswana” AND “Australia Antigen” on PubMed resulted in one additional relevant article being retrieved. The same searches were conducted on Google Scholar, Semantic Scholar, and Consensus online platforms and no additional relevant articles were found.

A review of the 67 articles from the PubMed search for “Botswana” AND “hepatitis virus” according to the inclusion and exclusion criteria resulted in 39 articles being filtered out from the search of online platforms; 28 articles remained and were included in this review. The additional journal article found by searching for “Botswana” AND “Australia antigen” on PubMed as well as an additional pre-internet article were also included in the review. A total of 30 articles were thus included in the review.

## 4. HBV in Botswana

### 4.1. Epidemiology of HBV and HIV-HBV Co-Infection in Botswana

Since the year 2006, the prevalence and distribution of viral hepatitis in Botswana have been studied mainly in PLWH. A description of HBV-related research prior to 2006 and/or not related to HIV-HBV co-infection can be found in Appendix A. Most of these studies have been undertaken by the research group at the Botswana Harvard Health Partnership (BHP). Due to the mandate of the institute to study different aspects of HIV, study cohorts including participants who were living with HIV were accessible for viral hepatitis research. Table 2 summarises the HBsAg prevalence from studies conducted in Botswana thus far.

In 2006, Wester et al. investigated hepatitis among PLWH (N = 160) at Princess Marina Hospital (PMH) in Gaborone. They reported an HBsAg prevalence of 10.6% and an anti-HBc prevalence of 58% [32]. In 2011, Patel et al. performed a retrospective review of charts of adults living with HIV (N = 266) who were parents or guardians of patients in the Family Model Clinic (FMC) of the Botswana-Baylor Children’s Clinical Center of Excellence (BCOE) in Gaborone. They found an HBsAg prevalence of 5.3% and an anti-hepatitis C IgG prevalence of 0.8% [33].

The BHP Bomolemo cohort (2008–2011), evaluating the efficacy of Truvada (TDF and emtricitabine-FTC) in cART treatment-naïve PLWH, was then investigated for HBV. Approximately 9.3% of the 300 participants were positive for HBsAg [41]. A 2017 study by Mandiwana and Tshitenge focused on HAART-eligible participants (N = 118) from mainly rural locales across the central region of Botswana and found an HBsAg prevalence of 5.1% [35]. Subsequently, the incidence of HBV in the BHP Botsogo study cohort of HIV subtype C disease progression (2005–2009) was 22 incident HBV cases (inclusive of all follow-ups) among 435 participants [37]. The baseline HBV prevalence was 4.8% (21/435).

Next, the Botswana Combination Prevention Project (BCPP) (2013–2015) cohort consisting of 30 matched rural communities—15 with enhanced HIV interventions and 15 with standard-of-care treatment—yielded extensive information about HBV. The HBsAg prevalence was 8% among 3304 participants living with HIV. Approximately 56% of 3218 PLWH had positive anti-HBc serology, indicating previous exposure to HBV or an ongoing HBV infection. The overall HBeAg prevalence of the cohort was 11%. Additionally, 10 out of 11 highly viraemic participants living with HIV were positive for HBeAg and were viraemic despite LAM- or TDF-containing treatments. Of the viraemic, HBeAg-positive participants, four were positive for anti-HBc IgM, indicating an active HBV infection. These results, in a large cohort of PLWH, showed that there was potentially a similar pattern of HIV-HBV co-infection within the country, and that HIV cART was not effectively treating HBV infections in all cases [40]. Interestingly, communities in the northwest of Botswana with the highest anti-HBc prevalence did not have higher HBsAg or HIV prevalence, suggesting increased exposure to HBV that was not in tandem with HIV [40].

Since the late 1990s, OBI has been recognised as a challenge to the accurate reporting of HBV prevalence and management [42]. The inability to detect OBI using routine serological testing—the only HBV testing performed in most locales—means that the prevalence of HBV is likely underestimated. Individuals with OBI may have reactivation of HBV that is unknown, particularly when they are immunocompromised, as in the case of HIV-HBV co-infection, complicating their clinical management. Individuals with OBI would risk not being treated and potentially have OBI-associated drug resistance when they are treated due to mutations specific to the OBI phenotype [40].

The Bomolemo cohort was used to investigate OBI for the first time in Botswana. Among the participants who were HBsAg-negative, 26.5% were HBV DNA-positive, indicating OBI [43]. An OBI prevalence that is more than twice the HBsAg positivity rate is concerning, as OBI testing is not routinely performed. The OBI prevalence is similarly as high as prevalences found elsewhere in sub-Saharan Africa [44]. An investigation of samples from the Botsogo [45] and Dikotlana [46] studies on the natural history of HIV found an OBI positivity rate of 14.7% (11/76) in baseline samples with sufficient plasma for HBV viral load testing. Analysis in 2- and 3-year follow-up samples with sufficient plasma volumes found an incident OBI positivity rate of 26.2/100 person-years. Incident OBI cases were more frequent among men (61.1%) and among participants with CD4^+^ T-cell counts ≤450 cells/mL (*p*-value = 0.02). It is possible that many of the incident OBI cases (55.9%, 19/34) were reactivations, as they were previously anti-HBc-positive [47]. Additionally, 381 HBsAg-negative participants from the BCPP cohort were screened for OBI, and 126 (33%) had detectable HBV DNA. Of 118 of these individuals, 67 (56.8%) were positive for anti-HBc and 51 were serologically negative OBI cases—13.4% of the initial 381 HBsAg-negative participants. Using HBV screening guidelines for PLWH, the 51 serologically negative OBI cases would not have been identified [40].

HBV is often transmitted vertically. Thus, pregnant women are an important demographic to monitor for changes in HBV viral markers. Matthews et al. investigated HIV-HBV co-infection in 950 pregnant women from Botswana and South Africa, as well as HBV infection in 72 HIV-negative pregnant women. The Botswana cohort was drawn from the BHP MmaBana study (2006–2008) and comprised 443 women, 17 (3.1%) of whom were HBsAg-positive. Of 60 HBsAg-positive participants, 16 were HBeAg-positive, indicating an overall rate of 26.7% with active HBV replication. Ten of these participants were from the MmaBana cohort; two (20%) were HBeAg positive. A similar percentage of HBeAg-negative participants 30% (9/30) had HBV viral loads above 2000 IU/mL, values congruent with actively replicating HBV infection that qualifies for treatment using current WHO guidelines. This finding suggests that although HBeAg is a useful marker for HBV staging, it should be considered together with HBV viral load for optimal results. There was no significant association found between HBV status and CD4^+^ T-cell count or HIV viral load in the Botswana participants; however, a lower CD4^+^ T-cell count was observed in HIV-HBV co-infected South African participants [34]. The BHP Tshipidi study cohort (2010–2012) of pregnant women living with/without HIV in Botswana reported lower HBsAg (2.1%) (N = 752) and OBI (6.6%) prevalences than prior studies. A higher HBsAg prevalence was found in those also living with HIV 3.1% compared to 1.1% in those who were HIV-negative, although this difference was not statistically significant [36]. Three women in the Tshipidi cohort—who were not living with HIV—had positive HBeAg serology. Detectable HBeAg serology, particularly in HIV-negative participants with high HBV viral loads, is cause for concern due to the potential of transmission to their babies as well as not having the benefit of dual/cross-treatment offered by HIV cART [36]. Further analysis of the Tshipidi cohort found no association between maternal HBV infection (HBsAg^+^ and OBI) and preterm birth (<37 weeks), stillbirth, low birth weight (<2.5 kg), and infant hospitalisation by 24 months [48].

Two studies conducted in Botswana have examined HBV prevalence in infants. A 2020 study found no HBsAg among 304 infants and a HBsAg-positive prevalence of 1.74% in mothers in the BHP Mpepu study cohort which looked at the effect of co-trimoxazole on mortality in HIV-exposed but uninfected children in Botswana (2011–2015) [38]. Infant dried blood spots (DBSs) from the Mpepu study were utilised to investigate anti-HBs titres. Anti-HBs titres were lower in infants living with HIV versus HIV-uninfected infants (74.5% versus 89.2%), indicating a possible decrease in the protective effect of the HBV vaccine due to HIV-HBV co-infection. Only three (0.6%) infants were HBsAg-positive, indicating that despite the incomplete vaccination protection of all infants, HBV transmission events were rare [39].

An atypical HBsAg-positive but anti-HBc negative phenotype (HBsAg^+^/anti-HBc^−^) was reported in BCPP participants [49]. An anti-HBc positive result is expected in the case of an HBsAg-positive result, but the HBsAg^+^/anti-HBc^−^ phenotype has been reported as summarised by Phinius et al. [49]. Differences in test kit sensitivities have been shown to affect the prevalence of the HBsAg^+^/anti-HBc^−^ phenotype. The proposed reasons for the true HBsAg^+^/anti-HBc^−^ phenotype include immunosuppression and mutations in the HBV core region [49]. Among BCPP participants, 13.7% (N = 263) tested positive for HBsAg and negative for anti-HBc. These participants were younger, female, cART-naïve, and had a detectable HIV viral load compared to those with HBsAg^+^/anti-HBc^+^ serologic findings. There was no statistically significant difference in mutations between the HBsAg^+^/anti-HBc^+^ and HBsAg^+^/anti-HBc^−^ groups [49]. The reasons for this phenotype continue to be investigated.

There is substantial variability in prevalence estimates across cohorts, attributable to numerous factors. The studies are compiled over several decades, wherein testing modalities have changed which may have an effect on results. The studies were conducted in cohorts with different demographics and objectives. Many of these cohorts were not initially designed considering HIV-HBV co-infection. This resulted in missing data. An example of instances where data was missing is in the lack of the full complement of serological tests needed to determine the stage of infection, as well as OBI and prior infection rates, important for determining AHB or CHB prevalence. Participants’ prior HIV cART status varied between studies, as did access to cART, according to demographic and policy differences in who HIV cART is made available to over time. Pregnant women, for example, have mandatory screening and at times improved access to treatment for infectious diseases and cART initiation policies have changed from viral load and CD4^+^ T-cell count-based treatment plans to availability for all individuals found to be living with HIV. These different factors should be considered when planning future HIV-HBV co-infection research.

### 4.2. HBV Molecular Findings in Botswana

The molecular biology of HBV is important to its infectivity and virulence. The different genotypes, sub-genotypes, and quasi-species of HBV confer different phenotypes which cause varied clinical outcomes. There are nine different HBV genotypes named A-I and a putative J genotype [50,51]. Genotypes A, D and E are predominant in Southern Africa. Genotypes have more than 8% variation from each other [50,51]. There are also more than 40 subgenotypes differing between 4 and 8% at the nucleotide level [50,51,52]. HBV genotypes are thought to result from different introductions of the virus into human populations and from the spread of these genotypes along migration patterns after introduction [53]. Different HBV sub-genotypes and quasi-species are thought to occur due to HBV’s unusual, error-prone replication through RNA intermediates using viral POL lacking proofreading ability in addition to natural selection and host immune pressure [53,54]. Different HBV genotypes are associated with different clinical progression and outcomes [53,54]. For example, HCC is associated with an earlier onset in the case of HBV A1 circulating in southern Africa, E found in West Africa and F in South America and the Arctic Circle [54]. Subgenotype A1 is the most prevalent HBV genotype that circulates in Botswana with D and E also being found in the population [55].

In 2015, the first study of circulating HBV genotypes in Botswana was conducted in pregnant women living with HIV. Genotype D—the D3 subgenotype—was most common. The HBV polymerase (*Pol*) gene was sequenced in 16 participants with detectable HBV viral load. RAM—M204I was observed in one individual [34]. A subsequent study included HBsAg-positive samples retrieved from several cohorts—Bomolemo, MmaBana, Botsogo, Basadi, and Tshepho—of ART-naïve PLWH at the BHP. A 415-base pair (bp) fragment of the HBV *surface/Pol* gene was sequenced to determine circulating HBV genotypes and the presence/absence of RAMs. Sub-genotype A1 was most common (80%), while D3 (18.6%) and E (1.4%) were also present [56]. Twelve individuals had vaccine escape mutations; however, there were no RAMs. There was also no significant association of the mutations or genotypes with measures of liver disease such as the liver enzymes alanine aspartate aminotransferase (AST), alanine alanine aminotransferase (ALT) or the AST to platelet ratio index (APRI).

Subsequently, a study of pregnant women from the Tshipidi cohort found a sub-genotype distribution of 45.5% for both A1 and D3 and 9% for genotype E. Vaccine escape mutations and RAMS were not analysed in this cohort [36]. In the Mpepu study, 5 mothers were HBsAg positive. 4 were infected with HBV sub-genotype A1 and one with genotype E [38]. Shaver et al. utilised DBSs from the Botswana National Antiretroviral Program and found that two HBsAg positive cases were infected with sub-genotype D3 [39].

In blood donor samples from the National Blood Transfusion Service (NBTS) (2014–2015), the sub-genotype distribution among HBsAg-positive individuals was 36.2% A1, 2.9% D2, and 58.3% D3. Mutational analysis in the blood donor group as well as in a chronically HBV-infected group that included participants from the Bomolemo, MmaBana, Botsogo, Basadi, and Tshepo studies found vaccine escape mutations in the S ORF of both the blood donor and the chronically infected HBV group [56]. Mutations associated with undesirable effects, such as impaired HBsAg antigenicity (surfaceD144A), immune and vaccine escape (sD144A, sY134H secondary to reverse transcriptaseV142A), HBV reactivation (sY134H and sD144A), and OBI status (sP120L) were specifically found in blood donors, who would not be exposed to any HIV cART that might be effective against HBV [57]. Analysis of full-length genomes from the Bomolemo and Tshipidi studies revealed 48% for both sub-genotypes A1 and D3 and 4% with genotype E. The genotype distribution was similar in HBsAg-detectable HBV-infected and OBI participants, suggesting that genotype is not associated with OBI status. Forty-three OBI-specific mutations were identified, of which 26 had not been reported previously in GenBank [58].

In the BCPP cohort, 100 of 107 (93.5%) sequences were genotype A1, 2.8% were D3, and 3.7% were E. Thirteen vaccine escape mutations were observed in 20% (18/90) of samples, with sK122R being the most common, although no vaccine escape mutations were associated with OBI. Mutations were also assessed using online tools (PROVEAN) for their effect on protein function, and the OBI-associated mutations threonine–proline Q6H, sV194A and precoreW28L were determined to have a deleterious effect [55]. Ninety-eight samples were assessed for RAMs. RAMs found were mainly against LAM (at genome positions 204, 180, and 173). The triple mutation rtM204V/L180M/V173L was predominant. Multiple significant mutations confer a greater risk of more pronounced resistance to cART. Interestingly, more RAMs were observed among individuals with a lower HBV viral load [55,59].

In silico analysis of OBI-associated mutations found 43 OBI-associated mutations within HBV sequences from Botswana. Of these, 26 were predicted to be deleterious and affect protein function. Functional analysis of these mutations would further elucidate their effect [60].

Molecular analysis of HBV in Botswana, particularly in the context of co-infection with HIV, has provided important insights into the circulating genotypes and common clinically relevant mutations present in Botswana. Continued investigation of viral genotypes, RAMs, vaccine escape mutations, and OBI-associated mutations present in Botswana can direct future research towards the effective therapeutic management of HBV. Table 3 summarises the main mutations found in Botswana cohorts in both HIV-HBV co-infected and HIV mono-infected cohorts.

### 4.3. HBV Host Genetics and Immunology Research in Botswana

Host factors are an important element in the pathogenesis of HBV. An effective immune response to HBV is mainly mediated by the adaptive immune response, primarily through CD8^+^ cytotoxic T-cell responses, and B-cells via antibodies, although interferon-γ (IFN-γ) is involved in early HBV infection [61]. CD8^+^ T-cells are known to frequently become exhausted during viral infection [62]. In relation to HBV, studies have shown that exhausted HBV-specific CD8^+^ T-cells have decreased cytokine and cytotoxic effects [63]. These T-cells also express the immune inhibitory receptor programmed cell death protein-1 (PD-1) amongst other inhibitory receptors. PD-1 binds to its ligand programmed death-ligand-1 (PD-L1) on cells, including hepatocytes [63]. The interaction of PD-1 and PD-L1 results in inhibition of the adaptive immune response, including to HBV. HBV-specific CD8^+^ T-cell exhaustion has been found to occur more often in the case of high viral loads [63,64]. B-cells can also become exhausted during HBV infection, resulting in impaired antibody production [65]. Persistent high levels of HBsAg secretion result in expression of dysregulated B-cells which express less anti-HBs and persistent anti-HBc, which is indicative of CHB [65]. These B-cells also express immune-inhibitory receptors such as PD-1 [65].

Human leukocyte antigens (HLAs), particularly HLA class I on the surface of host cells, are important to the adaptive immune response [61]. Genetic differences in HLA can contribute to the effectiveness of the immune response to HBV [66]. HLA-related studies to elucidate genetic differences have been conducted in the Botswana context. A study investigating HLAs in the Botswana and South African population demonstrated an association of HLA-A and HBV markers of disease progression such as HBeAg and HBV DNA levels [67]. In silico analysis of the putative binding of HBV peptide epitopes utilising HLA class II alleles common in Botswana found that peptides bound differently to the alleles. When applied to vaccines, the findings suggest that multi-epitope vaccines derived from different genotypes would be advantageous over broad-potency vaccines that include multiple epitopes from specific genotypes [68].

Beyond the genetic analysis of HLA markers, host genetic and immunologic research has not been conducted in Botswana and is a key area for future research for a complete understanding of HIV-HBV pathogenesis in the country. Participant demographics, including tribal and physical location, should also be included for a more comprehensive analysis.

### 4.4. HIV-HBV Co-Infection Response to HIV cART

An understanding of how HIV-HBV co-infection responds to treatment is essential for the clinical management of HBV. Tenofovir-containing HIV cART, which is also effective against HBV, is the standard treatment available and prescribed for HIV-HBV co-infection in Botswana [69]. The majority of people on HIV cART receive their medications through government programmes, although tenofovir-containing cART is also available in the private sector.

Findings from a study by Anderson et al. that aimed to determine the response to TDF and FTC combination (Truvada) first-line cART in HIV-HBV co-infected versus PLWH only indicated that HIV-HBV co-infected participants had worse immunologic outcomes compared to PLWH only [22]. In particular, a slowed CD4^+^ T-cell gain in co-infected participants was observed in the study. As higher CD4^+^ T-cell counts are associated with greater HBV DNA suppression over time, lower CD4+ counts and reduced CD4+ T-cell responses could also have affected HBV-related clinical outcomes. Despite this, HBV DNA levels generally reduced over time in the study [22]. As mentioned previously, in the BCPP cohort, HIV-HBV co-infected participants with positive HBeAg serology were still viraemic following LAM- or TDF-containing treatments; 36% of them were anti-HBc IgM-positive, indicating acute HBV or reactivation of HBV. The loss of HBsAg and the suppression of cccDNA are the hallmarks of HBV functional cure. In the Bomolemo cohort, HBsAg loss was 38%.

An investigation of HBsAg loss in PLWH on cART with CHB from the BCPP cohort found a 7.1% loss rate, with a higher rate among cART-naive participants [70]. This is similar to the HBsAg loss rates reported previously in PLWH [71,72,73,74] but lower than for the Bomolemo cohort. There was no association found between HBsAg loss and age, gender, HIV viral load, or CD4^+^ T-cell count [70]. HBsAg loss found in treated individuals in both the Bomolemo and BCPP cohorts as well as in prior studies is sub-optimal, a result that can be improved with research aimed at improving treatments.

Adherence to HIV cART was not investigated in these cohorts but is an important potential confounder, as non-adherence affects clinical outcomes. The duration on cART and therefore the potential for resistance mutations, resulting in worse clinical outcomes is another potential confounder that was not investigated.

## 5. Future Work and Perspectives

There is a need to conduct further laboratory-based and clinical research on HBV in Botswana’s general population to understand the epidemiological and viral features of HBV infection across the country. An HIV-negative/HBV mono-infected cohort is needed to better compare scientific findings related to HBV in the HIV-HBV co-infected population, as findings from cohorts of PLWH are not generalisable. HBV prevalence of 4% within HBV mono-infected individuals in the BCPP cohort [10] and 46.7% in liver disease patients (living with and without HIV) [75] indicates that HBV is present and needs to be addressed within the general population.

Epidemiological monitoring of disease is an important initial step for healthcare planning. Studies conducted in HIV-HBV co-infected individuals may not represent the general population. Monitoring of HIV-HBV co-infection should therefore be prioritised, including monitoring of the OBI prevalence in vulnerable groups, such as those living with liver diseases or HIV. This monitoring should be performed by the healthcare authorities in Botswana to gain more robust insight into the status of HIV-HBV co-infection in the country. HBsAg testing should continue for those suspected to have HBV and be made available country-wide to diagnose and treat those who test HBsAg-positive. Increased access to HBsAg testing will decrease the burden on the healthcare system as well as morbidity for those living with HBV, should they develop disease sequelae. OBI testing for the general population should also be in the pipeline for HBV management by the healthcare authorities in Botswana. Pregnant women could be prioritised for prevention of mother-to-child transmission of HBV through testing, treating and monitoring efforts. Importantly, the infrastructure that is already in place for HIV screening and monitoring can be leveraged for HBV diagnosis and treatment as well, including for people not living with HIV, reducing the investment of scaling up. Babies in need of HBIG in addition to an HBV vaccine birth dose could also be identified during pregnancy. HBV vaccination is now recommended for pregnant women who are HBsAg-negative in order to prevent them from acquiring HBV in the United States of America [76]. A similar recommendation would be beneficial for Botswana. Efforts should aim to improve the HBV vaccine birth-dose uptake among infants. The prevalence of OBI in the HIV-HBV co-infected population in Botswana is estimated to be between 6.6% and 33% [55]. Combined with the estimated proportion of PLWH who are HBsAg-positive (1.1-10.6%) and the 4% in the general population, there may be a large number of people who could develop devastating CHB-related sequelae without treatment [8,10,70]. Financial resources should be allocated towards these fundamental preventative interventions as they are an important starting point towards curbing the prevalence of HBV in Botswana.

There are currently no guidelines for the management of viral hepatitis, including HBV, in Botswana. The lack of guidelines affects the diagnosis, treatment and monitoring of people living with HBV, as HCWs are left to make subjective decisions based on individual experience when managing their patients. Guidelines are needed to sensitise HCWs in primary care settings to the risk factors, signs and symptoms, as well as management processes for people living with HBV. The uptake of vaccines among adults may also increase once more HCWs are aware of the options available to their patients. The additions made to the Botswana 2023 integrated HIV clinical care guidelines are commendable, although room for improvement still exists [69]. A major omission is that only testing for HBsAg is performed in PLWH who are currently on triple-therapy HIV cART who are to be considered for a switch to dual-therapy regimens [69]. OBI cases would be missed in this population of people being switched, which represents the vast majority of PLWH, as the incidence of new HIV infections has greatly reduced in the past 15 years [8]. Additionally, HBV viral load testing could be included in the initial screening for new cART initiates and not only on follow-up visits. Despite the needed improvements there is promise for identifying, treating and monitoring many more people living with HIV-HBV co-infection and HBV mono-infection should the public healthcare system successfully implement the new HIV clinical care guidelines and pending viral hepatitis guidelines [69].

Results from the Bomolemo and BCPP cohorts where HIV viremia was still present support that continued research into the effect of first-line HIV cART on HIV-HBV co-infection is imperative. This is required for treatment guidelines as well as to optimise treatment for those living with HIV and HBV, with an aim of achieving HBV functional cure.

Continued research into the circulating HBV genotypes and mutations is needed. Genotypes have been associated with different clinical outcomes [77] and distinct mutational profiles [77,78]. This may require alterations to treatment and could lead to vaccine escape. Non-genotype-associated mutations, particularly those with the potential for clinical impact, including therapeutic resistance or vaccine escape, should also be monitored and novel ones characterised. The work on genotypes and mutations is a valuable addition to the body of work on HBV, particularly in sub-Saharan Africa. Additionally, the definitive findings from mutational research need to be applied in clinical settings by establishing drug resistance testing, similar to how HIV cART drug resistance-associated mutations are monitored in PLWH. This will allow for optimal HBV antiviral treatment.

HBsAg loss was investigated in the Bomolemo and BCPP cohorts, with 38% and 7.1% HBsAg losses, respectively, indicating a large potential for improvement. Uncovering the molecular predictors for HBsAg loss in the Botswana population will require further work in HIV-HBV co-infected as well as mono-infected cohorts. 

Further investigation into host factors involved in HBV suppression in the Botswana population and the region is needed. Continuation of the HLA research including sequencing and functional characterisation of HLA mutations is needed to verify the findings from in silico analyses, as well as to identify other beneficial or unfavourable HLA alleles related to HBV infection. Future research into therapeutic vaccines could build on knowledge of how the immune system interacts with HBV to mount an immune response, and how HLA differencess affect that response. Investigation of single-nucleotide polymorphisms (SNPs), as previously studied in Asian and other African populations [72], could also elucidate additionalgenetic differences that contribute to the immune response in the Botswana population. Functional characterisation and analysis into differences in both T and B lymphocyte (in particular for CD8^+^ cytotoxic T-cell) immune responses against HBV in HBV mono-infected and HIV-HBV co-infected Botswana cohorts is another avenue that may help elucidate differences in host responses to HBV infection [79].

A summary of the recommendations given in this paragraph can be found in Table 4.

There are several limitations in the data, which are discussed in more detail in the text. The variation in demographic and physical location of populations assessed for the review should be considered as a potential confounder due to differences in access to diagnosis and treatment of HBV, which may lead to over- or underestimation of HIV-HBV-related findings. The differences in demographics and location may also cause differences in HBV response to cART. HBV cART response may also be affected by non-adherence and duration of treatment, both increasing the chance of drug resistance mutations. Both factors are known to affect HIV response to cART and are not discussed in the articles. The lack of any OBI testing by healthcare authorities is an important point to re-emphasise, as the true burden of HBV cannot be assessed and managed without this information. Molecular analysis of the circulating HBV viruses in Botswana has been studied in a research setting but has not yet been implemented into the healthcare system; this is a gap to be filled in the future. The prevalence of HBV varies considerably between studies. The causes of this variation include changes in the test type employed; differences in demographics, as discussed above; the study design not being initially set up to investigate HIV-HBV co-infection; and changes to policy regarding access to cART. One of the biggest hurdles to HBV management is the lack of viral hepatitis guidelines for Botswana. Definitive guidelines are the first step in bringing objective, equitable quality healthcare to those living with HBV in Botswana.

Limitations to the scoping review process include lack of access to certain government reports, such as the one on the prevalence of HBV in 1994, as well as defined dates for HBV birth dose vaccination introduction. Access to and verification of articles was not a hinderance, as most of the work was conducted at the BHP or by collaborators.

## 6. Conclusions

The HBsAg prevalence among PLWH in Botswana has been reported as high as 10.6%, with OBI prevalence reaching 33%. These statistics translate to a potentially extensive disease burden. The effect of this HIV-HBV co-infection is varied, with higher HIV viral loads and lower CD4^+^ T-cell counts seen in the HIV-HBV co-infected group as compared to the HIV only group in some studies and no statistical significance found in others. However, two studies with HIV-HBV co-infection treatment data have found sub-optimal control of HIV disease with tenofovir and/or LAM in HIV-HBV co-infected participants, suggesting a negative clinical effect of co-infection.

Research into HBV in Botswana has revealed that the main genotypes prevalent in Botswana are A1 and D3, as well as genotype E, though the latter is found in lower proportions. Multiple RAMs, vaccine escape mutations, and OBI-associated mutations have been detected in the Botswana population. These mutations should be monitored going forward to allow for effective genotype and mutation-based management.

Botswana is outperforming most African countries in infant HBV vaccination coverage, with an estimated 95% coverage versus 76% across the rest of Africa [80]. However, HBV timely vaccine birth dose coverage in Botswana is only 56%, according to WHO immunisation data [14] the shortfall of which is an opportunity for vertical transmission to continue. The most recent birth rate statistics from 2023 indicate that there were 46,352 births in that year [81] 56% of which is 25,957. The total population of Botswana is 2,359,609, according to the most recent population census from 2022 [82]. This translates to just under 1% of the population each year not receiving a timely HBV birth-dose vaccine.

Guidelines for the management of HBV are needed to direct HBV-related healthcare for the general population, including adult vaccination against HBV. Relatedly, a promising step is the inclusion of multiple HBV screening tests for PLWH in the 2023 Botswana HIV clinical care guidelines, which will hopefully increase identification and treatment of HBV in this cohort.

Further work on HBV-related research is ongoing, including the addition of research cohorts including people who are living with HBV and not HIV. This research, combined with steady investment in all aspects of HBV management by the Botswana government, lays the foundation for reducing the incidence of HBV in the country in the coming years.

## Figures and Tables

**Table 1:** Summary of HBV epidemiology, diagnosis, vaccination and treatment.

HBV Background	Summary	References
Epidemiology	Worldwide, WHO statistics estimate that there are 254 million individuals living with HBV infection, with most living in Africa and Asia. The 2030 WHO elimination targets for HBV are for 90% of people to be diagnosed and for 80% of those diagnosed to be receiving treatment. HBV shares the same transmission routes as HIV and is a concern in populations with a high HIV disease burden, such as the population in Botswana. Globally, it is estimated that 1% of people living with HBV are also living with HIV. A meta-analysis from 2019 estimates the global prevalence of HIV in people living with HBV to be 7.6% (2.7 million people, 69% of whom are estimated to live in Africa). In Botswana, the prevalence of HBV in PLWH ranges from 1.1% to 10.6%.	[1,4,9,10,11,12]
Diagnosis	According to the WHO, HBV positivity should be determined by a positive HBV surface antigen (HBsAg) result. Other serological tests, such as HBV core antibody (anti-HBc), HBV surface antibody (anti-HBs), and HBV “e” antigen (HBeAg), can also be performed to understand disease chronicity. The WHO also recommends that HBV DNA be quantified in individuals living with HBV for treatment planning, with an HBV DNA level of >2000 IU/mL (~10,000 copies/mL) set as a treatment threshold.	[3,12]
Vaccination	The HBV vaccine is given as a schedule of at least three doses, and up to four in infants. In infants, the first dose is recommended as a birth dose within 24 h of birth to reduce the risk of vertical transmission, with two or three additional doses given in the first year of life. HBV immunoglobulin (HBIG) therapy is a solution of high-titre anti-HBs purified from the serum of people immune to HBV and is recommended to be administered to prevent HBV infection within 24 h of HBV exposure. Vaccine escape mutations are a concern for vaccination as the virus evolves. Records of infant HBV vaccination in Botswana can be traced back to 2000, although vaccinations have been stated to have begun in the late 1990s. The infant birth dose coverage for Botswana stands at 56%. In the Botswana public health system, HBV vaccination in adults is generally limited to those who may come into contact with infected blood, such as healthcare workers (HCWs), although deficits persist even in HCWs.	[12,13,14,,15,16,17,18,19,20]
Treatment	NRTI’s, particularly tenofovir (tenofovir disoproxil fumarate [TDF] or tenofovir alafenamide [TAF]) or entacavir (ETV), have potent antiviral effects. Tenofovir has a high genetic barrier to HBV (and HIV) resistance. HBV drug resistance-associated mutations (RAMs) that would reduce or negate tenofovir’s antiviral effect emerge infrequently. Tenofovir can also be used to treat HIV and is included in many HIV regimens in Botswana. In 2020, the WHO recommended that TDF be given prophylactically to pregnant women to prevent transmission of HBV to their infants. In Botswana, prophylactic TDF is not currently prescribed specifically for all pregnant women, although pregnant women living with HIV may receive TDF as part of their HIV treatment.	[3,4,12,21,22,23]

**Table 2:** Summary of HBsAg prevalence findings in Botswana over time.

Publication Year	Investigators	Population	Number of Participants	Method	HBV Prevalence
2006	Wester [32]	Adult PLWH at PMH	160	HBsAg ELISA	10.6%
2011	Patel [33]	Members of family model clinic at Botswana-Baylor Clinical Centre of Excellence	266	HBsAg ELISA	5.3%
2015	Matthews [34]	Pregnant PLWH HIV-negative pregnant women	HIV pos: 950 TOTAL = 443 Botswana (Gaborone) + 507 SA HIV neg: 72-SA	Botswana: HBsAg ELISA SA: HBsAg enzyme immune assay (EIA)	Botswana = 3.8% SA HIV pos = 9.7% SA HIV neg = 8.3% Botswana + SA HIV pos + SA HIV neg = 7.0%
2016	Anderson [22]	Adult PLWH (Bomolemo study)	300	HBsAg ELISA	9.3%
2017	Mandiwana and Tshitenge [35]	Adult PLWH	118	HBsAg ELISA	5.1%
2018	Mbangiwa [36]	Pregnant PLWH HIV-negative pregnant women	752 TOTAL= 384 HIV pos + 368 HIV neg	HBsAg ELISA	HIV pos = 3.1% HIV neg = 1.1% HIV neg + HIV pos = 2.1% OBI = 6.1%
2020	Phinius [37]	Adult PLWH (Botsogo study)	435	HBsAg	Baseline Prevalence = 4.8% Incidence over 3 time points = 3.6/100 person-years
2020	Baruti [38]	Pairs of mothers living with HIV and infants exposed to HIV (Mpepu study)	304 Infants 287 Mothers	HBsAg ELISA	Infants = 0% Mothers = 1.74%
2022	Shaver [39]	infants living with HIV and HIV-negative infants	469	HBsAg ELISA	0.6%
2023	Phinius [40]	Adult PLWH (BCPP)	3304	HBsAg ELISA	8%

BCPP—Botswana Combination Prevention Project, ELISA—enzyme-linked immunosorbent assay, EIA—enzyme immune assay, HBsAg—Hepatitis B surface antigen, HIV—human immunodeficiency virus, PLWH—people living with HIV, PMH—Princess Marina Hospital, SA—South Africa.

**Table 3:** Commonly occurring HBV mutations found in HIV-HBV co-infected and HBV mono-infected individuals from Botswana research cohorts.

Mutation Type	Mutation (S)	Cohort	Effect on Protein Function	Study Population
**RAMs**				
NRTI	rtM204I	MmaBana		HIV-HBV co-infected
LAM	rtM204V L180M V173L	BCPP		HIV-HBV co-infected
**OBI-associated mutations**	tpQ6H sV194A preCW28L	BCPP	Deleterious	HIV-HBV co-infected
	cT142S tpE88R sS55P rtM250L	BCPP	Neutral	HIV-HBV co-infected
	sP120L	Blood donors		HBV mono-infected
**Impaired virion secretion-associated mutations**	sT114S sS114L sN146S sC147Y			HIV-HBV co-infected
**Vaccine and/or immune** **escape mutations**	sK122R sT118M sC121R sQ129C sG130C sN131T sT131N sC137I sC139R	BCPP		HIV-HBV co-infected, immune and vaccine escape
	sD144A sY134H (secondary to rtV142A)	Blood donors		HBV mono-infected, vaccine escape
	sG130R	Blood donors		HBV mono-infected, immune and vaccine escape
	sG130N sT140S	Various HIV studies, blood donors		HIV-HBV co-infected, failure of diagnosis, immunoglobulin therapy or vaccine escape
	sY100C sR122K sT123A sC124R T126N sQ129R sM133T sM133L	Various HIV studies, blood donors		HIV-HBV co-infected, failure of diagnosis
	sG119R sC124R sT126N	Various HIV studies, blood donors		HIV-HBV co-infected, failure of immunoglobulin therapy
	sT126N sQ129R sM133L sF134V	Various HIV studies, blood donors		HIV-HBV co-infected, vaccine escape
**HBV reactivation-associated mutations**	sD144A sY134H	Blood donors		HBV mono-infected

BCPP—Botswana Combination Prevention Project, HBV—hepatitis B virus, HIV—human immunodeficiency virus, LAM—lamivudine, NRTI—nucleoside reverse transcriptase inhibitors, OBI—occult hepatitis B, RAM—resistance associated mutation

**Table 4:** Summary of recommendations for HIV-HBV co-infection and HBV management in Botswana.

Recommendations	Summary
HBV serological testing and NAT	HBV serological testing, including antibody testing for those who test HBsAg-positive, along with nucleic acid testing (NAT), should be available in order to sufficiently diagnose, monitor and treat people living with HBV.
HBV OBI testing	In addition to routine testing, consideration of OBI, particularly in immunologically vulnerable groups like PLWH and transplant patients, should be considered.
HBV mono-infection	More research cohorts of people living with HBV and not HIV are needed to determine the prevalence in the general population. A prevalence of 4% was reported in one study using convenience sampling.
HBV birth dose vaccination	Botswana has achieved 53% coverage according to the WHO. Access and timely administration by HCW require improvement to align with the WHO hepatitis B elimination goals.
HBV adult vaccination	HBV vaccination has only focused on HCW. Vaccination needs to be extended to adults, particularly groups such as pregnant women and PLWH where a reduction in transmission would provide a great benefit. In general, the vaccine should also be available to those born after infant vaccination was introduced into the healthcare system. Uptake amongst HCWs also needs to be improved.
Clinical training for liver disease specialists	Only one hepatology specialist and four gastroenterologists currently reside in Botswana. Increased training of clinical specialists that are trained to manage people living with CHB and other forms of chronic viral hepatitis and sequalae such as liver cirrhosis and HCC is needed to deliver optimal care to these patients.
HBV prevention of mother to child transmission (PMTCT)	Women living with HBV should be identified, treated and monitored throughout their pregnancies as a first step to prevent mother-to-child transmission of HBV. Babies born to these mothers should be given the HBV vaccine as well as HBIG. The HIV PMTCT programme can be leveraged for the identification and treatment of mothers living with HBV.

## Data Availability

Data were derived from public domain resources. These data were derived from the following resources available in the public domain: https://pubmed.ncbi.nlm.nih.gov/, accessed on 2 April 2024 and 2 October 2025.

## Appendix A

### Appendix A.1. History of HBV in Botswana Prior to 2006

Prior to 2006, there were limited studies of HBV in Botswana. The earliest mention of viral hepatitis in Botswana was in the 1970s. First was a study from 1973 by Nurse et al. of non-venereal syphilis and Australia antigen in the San people of the central Kalahari game reserve which found that 14% (29/203) of those tested were positive for Australia antigen—the former term used for HBsAg [83]. Another study by Bersohn et al. in 1974 found an HBsAg prevalence of 9.1% (N = 4972) in people from rural areas of Botswana, 2.1% (N = 2362) in Batswana living in urban areas of South Africa, and 10.3% (N = 328) within the San population of Botswana [84]. In 1985, there was an outbreak of non-A, non-B hepatitis in Maun, a town in the North-West region of Botswana, in which the disease course was consistent with a water-borne hepatitis outbreak. A total of 273 cases were reported, and there was a positivity rate of 47% (28/60) HBsAg and 69% (37/54) anti-Hepatitis D virus within those tested [85]. Prior to the outbreak, there was 13.6% HBsAg prevalence and evidence of anti-HDV in the population [85]. Additionally, a 2011 article by Patel et al. mentions a 1994 government report that found an HBsAg prevalence of 12% in the population [33]. After these initial studies, HBV-related research in Botswana stalled until 2006.

### Appendix A.2. HBV and Non-HIV Disease in Botswana

HBV research in Botswana has focused largely on asymptomatic PLWH, with few studies looking at viral hepatitis in symptomatic individuals, including those not living with HIV. In a cohort of 328 people presenting with hepatitis-associated symptoms at the internal medicine clinic at PMH between February 2015 and July 2016, 46.7% were HBsAg positive. Additionally, 4.6% of HBsAg-positive participants were HDV antibody-positive and 4.3% were HCV antibody-positive. These results indicate that viral hepatitis infections could be a significant cause of liver-related morbidity [5].A recent analysis that utilised HIV-negative samples from the BCPP cohort indicated an HBsAg prevalence of 4% (N = 2135) [6,7].

Most of the HBV-related research in Botswana has focused on its relation to HIV infection due to availability of samples. As HBV affects the liver, its effect on clinical outcomes when occurring in the presence of other diseases is important to consider.

People undergoing haemodialysis are at risk of contracting blood-borne infections such as HIV and HBV due to the shared use of equipment. In Botswana, the prevalence of HBsAg positivity using a rapid test and anti-HCV antibodies was determined in 168 end-stage renal disease (ESRD) patient volunteers undergoing haemodialysis at Bokamoso Private Hospital and The Renal Care Institute in Gaborone. At baseline, five people had positive HBsAg serology and two were positive for anti-HCV antibodies. During the three-month period of study follow-up, two people seroconverted to HBsAg positivity while 1 converted anti-HCV seropositivity. Although the seroconversion rate was considered low, the study is indicative of the potential threat of dialysis with regard to HBV and HCV in these patients, despite measures such as strict hygiene practices, regular HBV and HCV testing and separate facilities for those living with HBV or HCV and also undergoing haemodialysis in these centres. This is especially true as HBV and HCV infection in this study were not significantly associated with other factors that are known to increase the chances of contracting HBV or HCV, including the duration of haemodialysis, history of invasive procedures, HIV status, frequency of hospitalisation, and the history and number of blood transfusions [8].

Table A1 summarises HBsAg prevalence findings in mono-infected cohorts in Botswana over time.

**Table A1:** Summary of HBsAg prevalence findings in mono-infection in Botswana over time.

Publication Year	Investigators	Population	Number of Participants	Method	HBV Prevalence
1973	Nurse [83]	San in central Kalahari region of Botswana	203	Serological testing	14% Australia Antigen/HBAg
1974	Bersohn [84]	Southern Africa	4972—Batswana in rural South Africa (SA) 2362—Batswana in urban SA 328—Central Kalahari and SA-Botswana border San	Counter-current immuno-electrophoresis or Complement fixation	Batswana in rural SA = 9.1%, Batswana in urban SA = 2.1%, central Kalahari and SA-Botswana border San = 10.3%.
1989	Byskov [85]	People showing symptoms of a water-borne hepatitis outbreak	60	HBsAg ELISA	47%
1994	Government Report [33]				12.5%
2021	Mahupe [86]	Patients undergoing haemodialysis at Bokamoso Hospital and the Renal Care Institute in Gaborone	168	HBsAg RDT	4.2%
2021	Souda [75]	Patients at PMH Internal Medicine Clinic with signs of hepatitis	328	HBsAg ELISA	46.7%
2024	Anderson [10]	Adult HIV-negative people (BCPP)	2135	HBsAg ELISA	4%

BCPP—Botswana Combination Prevention Project, ELISA—enzyme-linked immunosorbent assay, RDT—rapid diagnostic test, HBAg—Hepatitis B Antigen, HBsAg—Hepatitis B surface antigen, HIV—human immunodeficiency virus, PMH—Princess Marina Hospital, SA—South Africa.
